# Lead-free piezoelectrics: V^3+^ to V^5+^ ion conversion promoting the performances of V-doped Zinc Oxide

**DOI:** 10.1038/srep41957

**Published:** 2017-02-06

**Authors:** M. Laurenti, M. Castellino, D. Perrone, A. Asvarov, G. Canavese, A. Chiolerio

**Affiliations:** 1 Center for Sustainable Future Technologies @POLITO, Istituto Italiano di Tecnologia, C.so Trento 21, 10129 Torino, Italy; 2 Department of Applied Science and Technology, Politecnico di Torino, C.so Duca degli Abruzzi 24, 10129 Turin, Italy; 3 Institute of Physics, Dagestan Scientific Center, Russian Academy of Sciences, Yaragskogo Str. 94, 367003 Makhackhala, Russia

## Abstract

Vanadium doped ZnO (VZO) thin films were grown by RF magnetron sputtering, starting from a ZnO:V ceramic target. The crystal structure, chemical composition, electric and piezoelectric properties of the films were investigated either on the as-grown thin films or after a post-deposition rapid thermal annealing (RTA) treatment performed at 600 °C for different lengths of time (1 and 5 min) in an oxygen atmosphere. Substitutional doping of Zn^2+^ with V^3+^ and V^5+^ ions strongly deteriorated the hexagonal wurtzite ZnO structure of the as-grown thin films due to lattice distortion. The resulting slight amorphization led to a poor piezoelectric response and higher resistivity. After the RTA treatment, strong *c*-axis oriented VZO thin films were obtained, together with a partial conversion of the starting V^3+^ ions into V^5+^. The improvement of the crystal structure and the stronger polarity of both V^3+^ – O and V^5+^ – O chemical bonds, together with the corresponding easier rotation under the application of an external electric field, positively affected the piezoelectric response and increased conductivity. This was confirmed by closed-loop butterfly piezoelectric curves, by a maximum *d*_*33*_ piezoelectric coefficient of 85 pm·V^−1^, and also by ferroelectric switching domains with a well-defined polarization hysteresis curve, featuring a residual polarization of 12.5 μC∙cm^−2^.

Wurtzite semiconductors^1^,^2^,^3^ are emerging as a promising alternative to the most common perovskite ferroelectric materials^4^ for the development of new generation, lead-free, semiconductor-based MEMS/NEMS technologies. Nowadays, perovskite lead zirconate titanate (PZT) still represent the best engineering solution due to a good piezoelectric response and ease of integration into micro/nano devices^5^. Despite such promising properties, PZT-based materials suffer from a major limitation, i.e. toxicity due to lead content. In the last years many governmental regulations have imposed restrictions on the use of toxic compounds. Therefore, there is a growing interest in looking for lead-free piezoelectric alternatives to PZT, that might give rise to a new generation of environmentally friendly piezo devices^6^.

Zinc oxide (ZnO) is currently one of the most investigated materials thanks to the presence of both semiconductor behavior and the piezoelectric effect, as well as biocompatible properties^7^. Several piezoelectric ZnO micro- and nano structures, such as compact^8^,^9^ and porous^10^,^11^ thin films, nanowires^12^,^13^, and nanorods^14^, can be easily obtained by several synthetic pathways^15^,^16^,^17^,^18^. These structures can be successfully exploited for the fabrication of both piezoelectric sensors and actuators, and for new generation energy harvesting systems. In order for ZnO piezoelectricity to take place the presence of a particular non-centrosymmetric crystalline framework, i.e. the hexagonal wurtzite structure, is required. When ZnO stabilizes in the wurtzite phase, the presence of a [001] preferential orientation along the *c*-axis direction is generally obtained. Hence, promoting the *c*-axis orientation in ZnO-based materials is fundamental. To this purpose, the sputtering technique is of great interest since it allows for the growth of strongly textured ZnO thin films in a very uniform and controllable way on wide-area substrates of different natures^8^,^9^,^19^. Moreover, sputtering is fully compatible with both semiconductor technologies and microfabrication processes.

ZnO is generally classified as an n-type semiconductor, since several electrically-active native defects, like oxygen vacancies and Zn interstitials, promote electrical conductivity. For this reason^8^,^9^, the resulting piezoelectric properties are generally lower than those theoretically predicted (*d*_*33*_ ~12 pm·V^−1^). However, the n-type nature of ZnO is advantageous for different applications, like the preparation of transparent conducting oxides (TCOs) and photoanodes for photovoltaic applications and solar cells^10^,^20^. In such cases, the electrical conductivity of pristine ZnO can be further enhanced by doping with selected elements as In^21^, Al^22^, and Ga^23^, which are the most widely diffused dopants for this purpose. The resulting doped ZnO samples feature electrical and optical properties which are fully comparable or even better than those exhibited by commercial TCO materials^24^.

On the other hand, doping can be exploited to partially compensate the n-type nature of pristine ZnO^11^,^25^. This leads to improved piezoelectric properties^26^ together with the appearance of ferroelectric phenomena^11^,^27^. Recently, different elements like Sb^11^, Fe^28^, Li^29^, Cr^30^ have been proposed for the synthesis of reliable p-type doped ZnO specimens. These have amplified piezoelectric coefficients as high as a hundred pm·V^−1^, making their piezoelectric performances comparable with those observed for sputtered PZT thin films^31^,^32^.

Another promising element is vanadium (V). Several works have reported improved piezoelectric properties of vanadium doped ZnO (VZO) nanomaterials associated with ferroelectricity. For example, VZO nanofibers synthesized by the electrospinning technique have been investigated^33^. In this work, as-grown nanofibers were found to be amorphous. However, after calcination was carried out at 700 °C for 1 hour, wurtzite structure crystallization occurred together with the insertion of the V dopants into the crystal framework. It was found that V doping induced a butterfly-shaped piezoelectric response together with a large piezoelectric coefficient *d*_*33*_ of 121 pm∙V^−1^. The improved piezoelectric properties were attributed to the switchable spontaneous polarization induced by the V dopants, i.e. the V–O bonds rotate more easily under the action of an external electric field. Nano-generation phenomena in two-dimensional VZO nanosheets (NSs) have also been exploited for the preparation of flexible nanogenerators, which showed an output current density of up to 1.0 μA·cm^−2^ under vertical compressive force^34^. Similarly, sputtered VZO thin films showed a giant piezoelectric coefficient *d*_*33*_ of 110 pm∙V^−1 ^35. Thin films were grown at 200 °C to promote the formation of the desired hexagonal wurtzite structure and the incorporation of V into the crystal cell, as witnessed by the shift of the corresponding (002) diffraction peak toward higher angles^36^. Also in this case the superior piezoelectric properties were ascribed to the emergence of switchable spontaneous polarization induced by the V^5+^ dopants combined with a relatively high permittivity. Again, the easier rotation of V – O bonds under the application of an external electric field was considered to be the microscopic origin for the observed phenomena. Even though different works have reported both the ferroelectric and piezoelectric properties of VZO, a clear and concise characterization of the ferroelectric one was missing until the experimental estimation of the Curie temperature for VZO nanorods grown using a low-temperature synthetic pathway (around 90–100 °C)^37^. In this case, a Curie temperature of 345 °C, together with a remnant polarization of 4.83 μC∙cm^−2^ and a coercive field of 5.43 kV∙cm^−1^, were determined.

It has further been found that V doping could also induce the enhancement of the electrical conductivity in VZO specimens. Hence, a reduction of the piezoelectric properties would be expected. This led to an increased electrical conductivity in VZO powders and thin films^38^,^39^, and the resulting materials were successfully exploited for the preparation of TCOs. High-temperature depositions (up to 500 °C) and post-deposition thermal treatments (up to 950 °C) were employed to promote the insertion of the V dopant and to specifically change the vanadium oxidation states. In particular, the existence of lower valence state V ions replacing Zn^2+^ in the host ZnO wurtzite structure was considered the main reason for the improved electrical conductivity of the doped specimens. Therefore, the chemical oxidation state of the V dopant seemed to play a fundamental role in determining the final electrical and piezoelectric behaviors of the investigated materials.

In the current work the influence of different vanadium oxidation states (V^3+^, V^5+^) on the piezoelectric properties of sputtered VZO thin films is investigated. V_2_O_5_ is not soluble in ZnO, as known from the equilibrium phase diagram of the two oxides^40^; nevertheless our materials are thermodynamically in a highly non-equilibrium state. This is true in each phase of the work: during SPS for the fabrication of the target (see further in the text Section 3.1), RF magnetron sputtering, and RTA. As-grown VZO samples showed a reduced crystallinity and the presence of a pseudoamorphous phase, together with the prominence of V^3+^ ionic species. All these aspects resulted in poor piezoelectric properties and reduced piezoelectric coefficients *d*_*33*_. After the RTA treatment the crystal structure of the annealed samples improved significantly, with a stronger *c*-axis texture. Moreover, a partial conversion of the starting V^3+^ into V^5+^ was obtained. These aspects positively affected the piezoelectric behavior of the annealed samples, which exhibited closed-loop butterfly curves. These aspects resulted in a further increase of the *d_33_* piezoelectric coefficient, which approached a maximum average value of about 85 pm·V^−1^.

## Results and Discussion

### Morphological and structural analyses of VZO thin films

**Figure 1:**
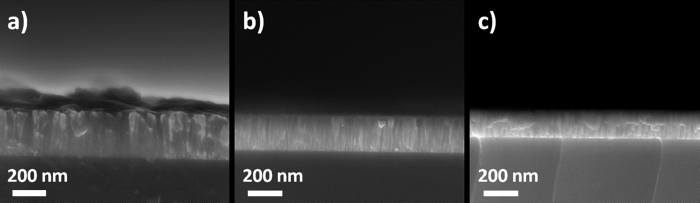
FESEM images showing the cross-section nanostructure of as-grown VZO thin films: (**a**) sample VZO-2%, (**b**) sample VZO-2.5%, and (**c**) sample VZO-3%.

Figure 1 shows the cross-section nanostructure of the as-grown VZO thin films. Independently of the deposition conditions, FESEM analyses point out the presence of closely-packed nanocolumns oriented perpendicularly with respect to the substrate. The average thickness changes from 180 nm for the samples grown with 10% O_2_ oxygen partial pressure ratio, to 220 nm for the samples grown with 5% O_2_, and finally to 285 nm for the samples grown using a pure Ar atmosphere. Besides the oxygen partial pressure ratio, all the growth parameters were kept constants. Hence, a slight decrease of the deposition rate occurred by increasing the oxygen content during the deposition.

**Figure 2:**
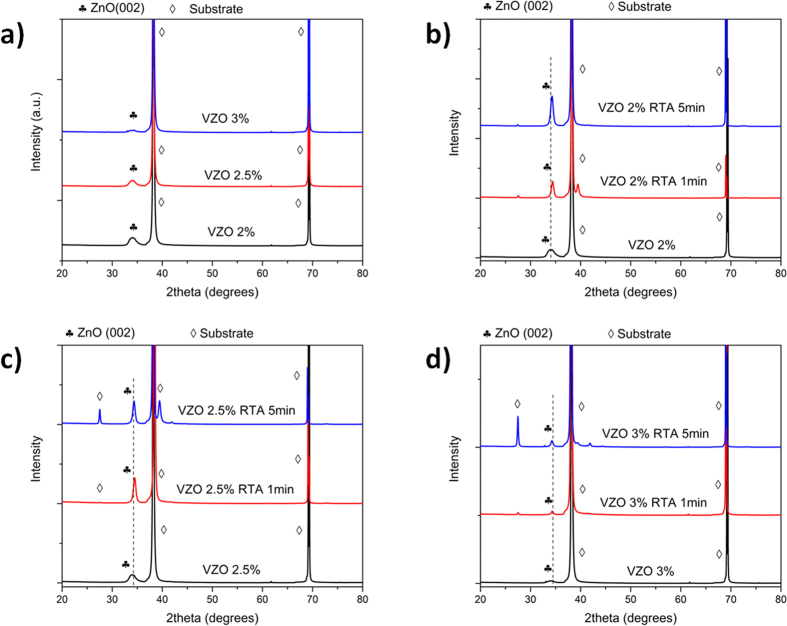
(**a**) XRD patterns of as-grown VZO thin films. (**b**) XRD patterns of samples VZO-2%, before and after the RTA treatment. (**c**) XRD patterns of samples VZO-2.5%, before and after the RTA treatment. (**d**) XRD patterns of samples VZO-3%, before and after the RTA treatment. The dotted lines represent the reference 2θ position for pristine ZnO sample.

**Figure 3:**
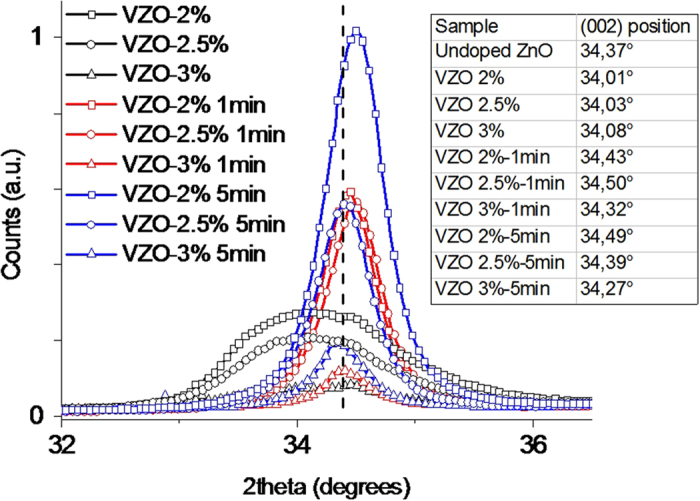
XRD patterns of VZO thin films before and after the RTA treatment, normalized with respect to the more intense diffraction peak (VZO 2%-5 min). Inset table summarizes the (002) peak position detected for each sample. The dotted line represents the (002) peak position assumed as reference in this work for undoped ZnO specimen.

XRD measurements shown in Fig. 2a evidence the effect of V incorporation on the crystal structure of the as-grown VZO thin films. Apart from the diffraction contributions coming from the substrate, a single (002) diffraction peak is detected for each sample on the whole 2θ acquisition range. This is attributed to the presence of the hexagonal ZnO wurtzite phase and of a slight crystal orientation along the *c*-axis direction. No additional contributions coming from metallic V or V oxides are detected. The weakness and broadening of the ZnO (002) peak, together with the strong left-shift of the corresponding diffracting angle (see Table 1 and Fig. 3), suggest that the crystal structure is notably deteriorated due to the growing presence of V dopant, which promotes at room temperature the formation of a pseudo-amorphous component in the thin film^41^.

After the RTA treatment the crystal structure is notably improved. Each annealed sample exhibits a single sharp and intense (002) diffraction peak, belonging again to the ZnO wurtzite phase (panels b, c, and d of Fig. 2). Depending on the considered sample, the (002) diffraction peak shifts toward different 2θ positions with respect to the corresponding one for pristine ZnO (34.37°) (see Supplementary Fig. S1). This effect points out the correct insertion of V in the host ZnO wurtzite structure and is due to the co-presence of V ions having different ionic radii that replace Zn^2+^ ions in the wurtzite cell. After the RTA process, the family of samples VZO-2% shows the most interesting crystal properties, with stronger and sharper diffraction peaks (Fig. 2b). In this case the (002) peak shifts toward higher 2θ angles after the annealing treatment. Concerning the family of annealed samples VZO-2.5% (Fig. 2c), after 1 min and 5 min annealing times the 2θ position is again shifted to higher angles in both the cases. Regarding the family of samples VZO-3%, the peak position is shifted to smaller angles (Fig. 2d). Moreover, the crystal quality is still generally reduced, as witnessed by the lower intensity of the corresponding diffraction peaks if compared to the diffraction contributions coming from the other annealed VZO thin films (see Fig. 3).

The improved crystal quality of the annealed samples is ascribed to the strong suppression of the crystal defects due to the thermal treatment. Moreover, the shift of the (002) diffraction peaks is related to the insertion of the V dopant into the wurtzite ZnO structure, and to the co-presence of V with different oxidation states and ionic sizes. In particular, it can be supposed that bigger V ions, involving a shift of the peak position toward lower 2θ angles, are mostly present for the family of samples VZO-3%. On the other hand, the right-shifted 2θ positions observed for the annealed samples of family VZO-2% and VZO-2.5% suggest that smaller V ions mainly influence the corresponding crystal structure and hence can more effectively participate to the doping process.

### Chemical composition analyses

**Figure 4:**
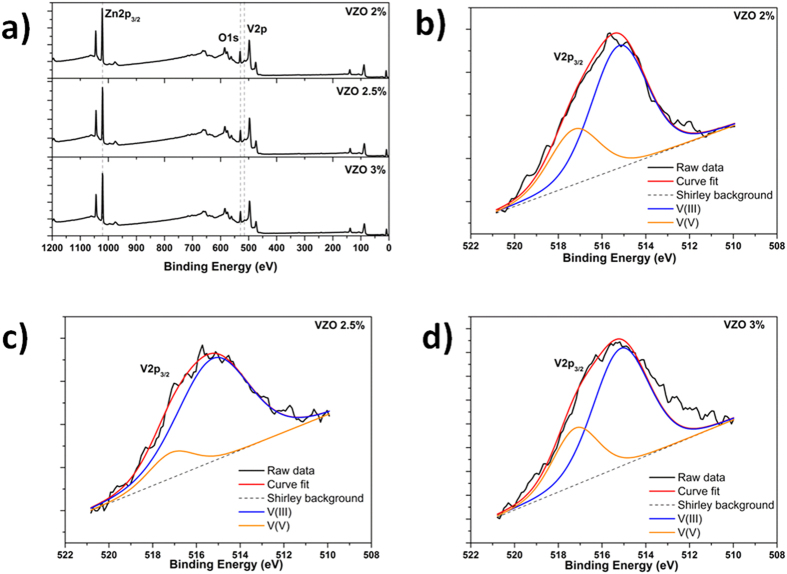
(**a**) Wide-scan XPS spectra of as-grown VZO thin films. (**b**–**d**) HR V 2p_3/__2_ XPS spectra for samples VZO-2%, VZO-2.5% and VZO-3%.

Figure 4a shows the wide-scan XPS spectra acquired for the as-grown VZO samples before undergoing to the RTA treatment. Besides Zn and O, the characteristic lines of V are detected as well, confirming the presence of the V dopant in each sample. The quantitative estimation of the relative atomic percentage for each detected element is summarized in Table 1. Among the different samples, no relevant changes are observed between the Zn at.% and O at.%, while the V at.% ranges between a minimum of 2% and a maximum of 3%.

In order to determine the V oxidation states in the as-grown VZO samples, high-resolution (HR) XPS spectra of the V 2p_3/2_ signal are acquired and shown in panels b, c and d of Fig. 4. The raw data are fitted by two components. The main contribution is positioned at 515.19 ± 0.05 eV and is ascribed to V^3+^ valence state ions involved in V_2_O_3_ chemical bonds^42^. The secondary peak, positioned at 517.21 ± 0.07 eV, is then associated to V^5+^ ions participating in V_2_O_5_ chemical bonds^43^. No additional contributions coming from V^2+^ and V^4+^ valence states are detected for any of the investigated samples. Therefore, their presence may be excluded within the detection limit of XPS.

Independently of the sample, the V dopant is mostly present in the V^3+^ valence state. However, some differences between the amount of V^3+^ and V^5+^ ions can be appreciated. Sample VZO-2.5% shows the highest amount of V^3+^ species at the expense of V^5+^ ions. On the contrary, the samples VZO-2% and VZO-3% show a higher amount of V^5+^ ions than the previous case, even though V^3+^ species are still predominant. The reason for a different quantity of V^3+^ and V^5+^ ions among the VZO samples is twofold. On the one hand the amount of V incorporated into the VZO layer, that may influence the valence state^11^,^28^. On the other hand, the addition of oxygen to the deposition atmosphere. In particular it is found that by increasing the amount of O_2_ into the deposition chamber from 0% up to 10%, V^3+^ first increases (sample VZO 2.5%) and then decreases (sample VZO 3%). This trend may depend on two competing effects. The first one relies on re-sputtering of oxygen atoms from VZO thin films when oxygen is added to the deposition atmosphere and reactive sputtering is performed^44^,^45^. In this case negative oxygen ions may be formed at the target surface. These are accelerated toward the substrate, inducing re-sputtering of oxygen (reducing conditions that promote the formation of V^3+^ species) and leading to the observed increase of V^3+^ amount at the expense of V^5+^ (sample VZO 2.5%). Then, when the highest amount of oxygen is introduced in the chamber (10%), oxidation conditions are more favorable and the amount of V^5+^ increases (sample VZO 3%).

HR XPS analyses of the V 2p_3/2_ signal are performed also for the ZnO:V target powders (see Supplementary Fig. S2). It is found that V is mainly present in V^3+^ (~60%) and V^5+^ (~18%) valence states, similarly to what is observed for the sputtered VZO thin films. The predominance of V^3+^ in the target material is due to reducing agents (hydrogen and carbon monoxide) that might be present during SPS target fabrication process, promoting the partial conversion of V_2_O_5_ precursor into V_2_O_3_^46^,^47^,^48^. V^2+^ and other nonstoichiometric oxide species are also present as minor components in the target. Therefore, it is presumable that such components could be present also on the sputtered samples in very little amounts, but falling under the detection limit of the XPS as mentioned above.

**Figure 5:**
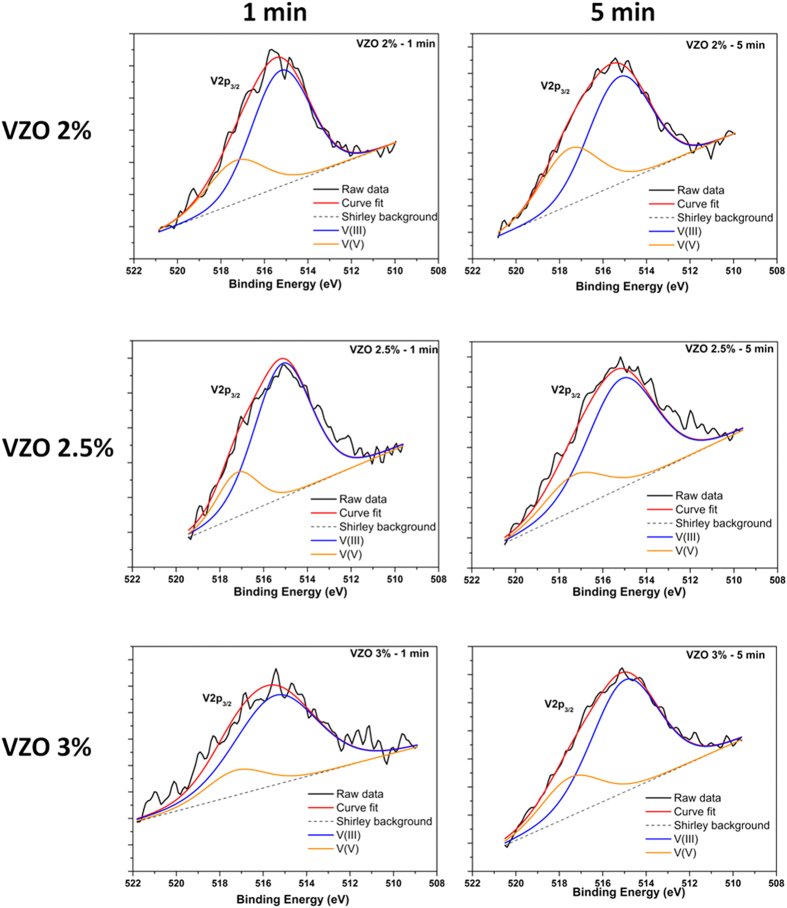
HR V 2p_3/2_ XPS spectra of VZO thin films acquired after the RTA treatment at different annealing times (1 min and 5 min).

In order to investigate the effect of the RTA treatment on the oxidation state of the V dopant, HR XPS analyses related to V 2p_3/2_ core-electron contribution are performed also on the annealed VZO samples (see Fig. 5). As visible from Table 2, a partial conversion of the starting V^3+^ ions into V^5+^ ones generally occurs after the RTA process for the family of samples VZO-2% and VZO-2.5%. On the contrary, the amount of V^5+^ ions decreases after annealing the sample VZO-3% for 1 min, and then increases after annealing for 5 min. This particular behavior may be due to the quite amorphous nature of the starting sample VZO-3% and to the consequent lower amount of grain boundaries, which generally act as preferential sites for oxygen adsorption^49^,^50^. These aspects initially prevent oxygen adsorption to drive the conversion of V^3+^ species into V^5+^. Therefore, after only 1 min, the effect of the annealing treatment on the starting V^5+^ species is only temperature-mediated, i.e. when V_2_O_5_ is submitted to high temperatures, it loses oxygen^51^ and the amount of V^3+^ species increases. However, as the RTA process time is increased, transition from a slight amorphous to a polycrystalline structure is observed in VZO thin films (see XRD patterns in Fig. 3). Once sample VZO-3% shows a polycrystalline structure, oxygen can more easily channel within the sample, effectively promoting the conversion of V^3+^ into V^5+^.

Nevertheless, the amount of V^5+^ species is found to be generally increased at the expense of V^3+^ ones. It is expected that substitution of Zn^2+^ (0.60 Å) with smaller V^5+^ ions (0.36 Å) induces the shift of the diffraction peak toward higher 2θ angles, while bigger V^3+^ ions (0.64 Å) should result into the shift toward lower 2θ values. Despite being six-fold coordinated, theoretical studies showed that V^3+^ may replace Zn^2+^ ions in wurtzite structures as well, although not occupying the same site^52^,^53^. Additionally, V^3+^ may also enter into the interstitial position of the ZnO lattice due to the similarity between ionic radii of V^3+^ (0. 64 Å) and the octahedral interstice of the wurtzite ZnO (0.61 Å)^54^. Therefore, XPS analyses agree with XRD results, that highlighted the shift of the (002) peaks toward higher or lower diffracting angles, dependently on the considered family of samples. Moreover, besides improving the crystal structure of VZO thin films, it is found that another effect of the RTA treatment is the partial conversion of the starting V^3+^ species into V^5+^ ions. This strongly influences the crystal structure of the annealed samples, as previously observed from the XRD characterization results, and is expected to influence the piezoelectric properties as well, as shown in the following section.

### Piezoelectric characterization

The piezoelectric behavior of VZO thin films is investigated by measuring the mechanical displacement (*D*) induced on the samples when applying an external bias voltage (*V*).

**Figure 6:**
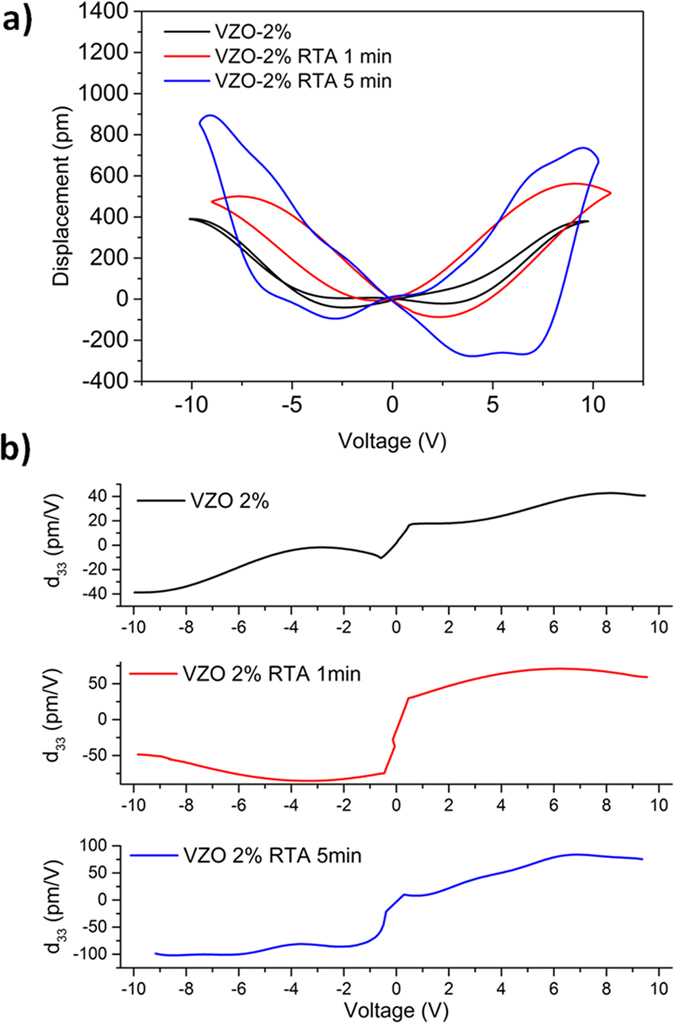
(**a**) *D-V* curves and **(b**) *d*_*33*_*-V* piezocoefficient for VZO-2% thin films, before and after the RTA treatment.

Figure 6a shows the *D-V* curves obtained for VZO-2% thin films, before and after the RTA treatment. All the samples show symmetrical butterfly closed loops, witnessing the presence of piezoelectric phenomena. Before the thermal treatment, the sample VZO-2% (285 nm) exhibits a maximum mechanical displacement (*D*_*pp*_) of around 400 pm. A general improvement is then obtained after the annealing treatment. In particular, after 1 min annealing time, the maximum *D*_*pp*_ of sample VZO-2% raises up to around 500 pm, and then remarkably increases till 900 pm after 5 min annealing time. The improvement of the piezoelectric behavior is highlighted by the corresponding *d*_*33*_*-V* curves as well. The as-grown sample already shows a remarkable increase of the piezoelectric coefficient (average *d*_*33*_ ~ 23 pm·V^−1^) than the pristine case (*d*_*33*_ ~ 12 pm·V^−1^). Then, it further increases after annealing the sample for 1 min (65 pm·V^−1^), reaching the maximum average value estimated in this work after 5 min annealing time (85 pm·V^−1^).

**Figure 7:**
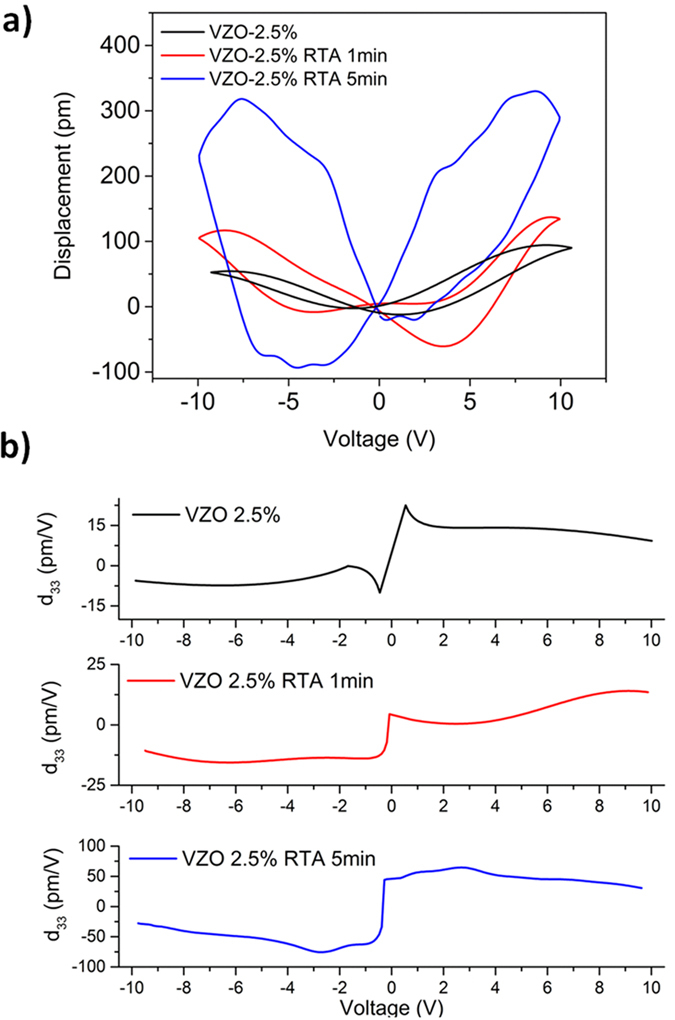
(**a**) *D-V* curves and (**b**) *d*_*33*_*-V* piezocoefficient for VZO-2.5% thin films, before and after the RTA treatment.

On the other hand, the samples VZO-2.5% (220 nm) show poorer piezoelectric response and piezocoefficient *d*_*33*_, as visible from Fig. 7. The as-grown thin film shows a weaker maximum *D*_*pp*_ (between 50 and 80 pm) and a limited *d*_*33*_ value (9 pm·V^−1^). After the RTA treatment, the piezoelectric behavior improves. In particular, after 1 min annealing time the *D*_*pp*_ slightly increases up to around 120 pm, while the corresponding piezocoefficient is still limited (10 pm·V^−1^). By increasing the annealing time up to 5 min, the maximum *D*_*pp*_ and *d*_*33*_ increase more prominently (300 pm and 51 pm·V^−1^, respectively).

**Figure 8:**
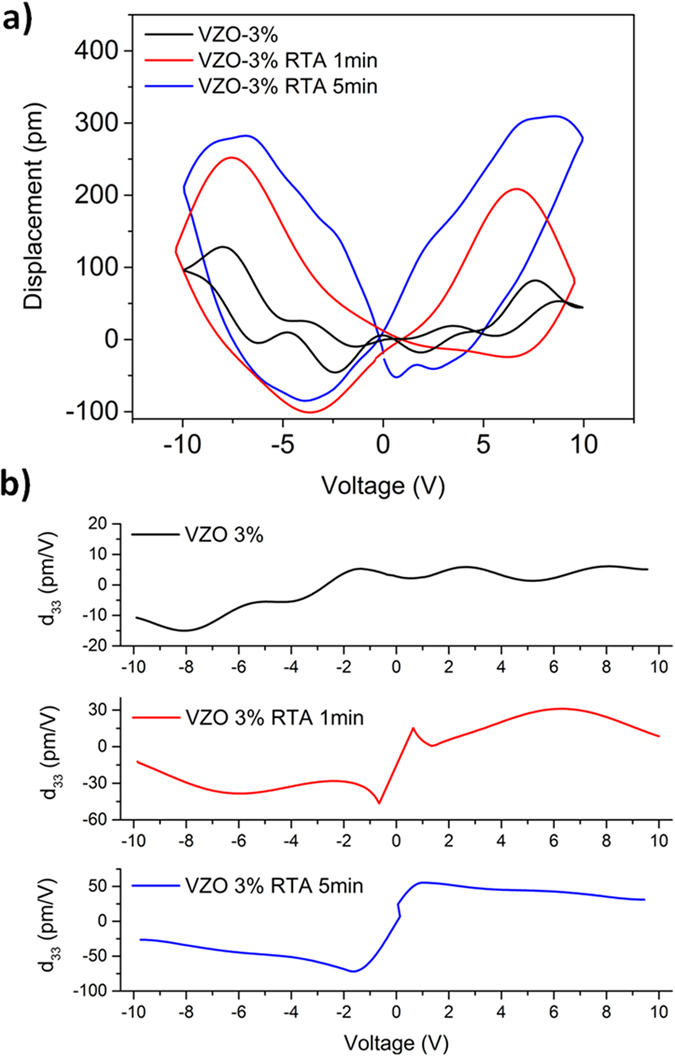
(**a**) *D-V* curves and (**b**) *d*_*33*_*-V* piezocoefficient for VZO-3% thin films, before and after the RTA treatment.

Figure 8a,b show the *D-V* and *d*_*33*_*-V* curves related to the family of samples VZO-3% (180 nm), respectively. A maximum *D*_*pp*_ of around 100 pm is obtained for the as-grown sample (*d*_*33*_ ~ 6 pm·V^−1^), which shows a quite unstable piezoelectric response against voltage variation. The piezoelectric behavior slightly improves after the RTA treatment also in this case. After 1 min annealing time, the maximum *D*_*pp*_ is increased (~200 pm) and the butterfly curve becomes more symmetrical and stable than for the as-grown sample (*d*_*33*_ ~ 25 pm·V^−1^). Then, after 5 min annealing time the piezoelectric displacement increases again, with a maximum *D*_*pp*_ of around 300 pm and a piezoelectric coefficient of around 44 pm·V^−1^.

The piezoelectric behavior of ZnO thin films is strongly related to the inter-lattice atomic distances among the Zn^2+^ and O^2−^ ions filling up the wurtzite structure. By properly changing such distances, the piezoelectric response can be tuned. A powerful tool to achieve such conditions is substitutional doping. This involves the replacement of Zn^2+^ ions in the wurtzite crystal cell with doping elements usually having different ionic radii and valence states than those of the substituted one (Zn^2+^, 0.60 Å). Both these factors finally result in a greater distortion of the crystal cell together with a stronger polarity of the chemical bonds within the wurtzite ZnO structure. However, additional crystal defects can be introduced if the lattice atomic distances are strongly modified. These result in a deterioration of the crystal quality of the doped specimens and consequently in the limitation of the piezoelectric response. Therefore, the variation of lattice distances and ionic valence states due to the doping process should be properly balanced in order for the improvement of the piezoelectric behavior to occur.

**Figure 9:**
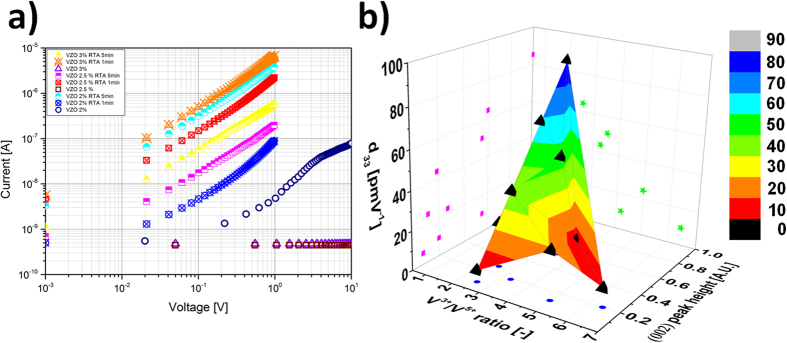
(**a**) IV curves in the logscale for the three families of samples. One point every 10 is shown for clarity. Less conductive samples were characterized in an extended voltage range. (**b**) phase space plot including the ratio between V^3+^ to V^5+^ V 2p_3/2_ peak collected after XPS analyses, crystallinity as (002) peak height collected after XRD analyses (Table 2) and *d*_*33*_; projections of experimental data on the three Cartesian coordinated planes are shown for completeness, behind experimental points (black tetrahedral) and color-mapped 3D surface.

In the particular case of vanadium dopant, several valence states and ionic radii hold^55^, each one being more or less different from that of Zn^2+^. Hence, the piezoelectric properties of the resulting V-doped ZnO thin films are strongly influenced by the chemical oxidation state of the introduced V dopant. In the present case XPS analyses reveal the presence of both V^3+^ and V^5+^ ions. In particular, samples VZO-2% and VZO-2.5% show an increased amount of V^5+^ ions at the expense of V^3+^ ones, together with a remarkable improvement of the corresponding crystal structure. Both these aspects are confirmed from XPS and XRD results, that also evidenced the shift of the (002) diffraction peak toward higher 2θ angles. The final result is the improvement of the piezoelectric behavior, especially for the annealed samples VZO-2% that indeed show the highest shift of the (002) peak together with the highest presence of V^5+^ ions. On the other hand, the strongly reduced piezoelectric behavior for samples VZO-3% is due to the mutual combination of a reduced crystallinity together with the increased amount of V^3+^ ionic species after the RTA treatment. The radius of V^3+^ ions and the corresponding valence state are slightly equal to that of Zn^2+^. These factors result in a very little distortion of the crystal cell together with a not remarkable change of the chemical bond polarity if compared to pristine ZnO. Both these aspects merge in a limited improvement of the piezoelectric behavior. Regarding the electrical properties, we computed resistivity (Table 2) from standard *I-V* curves (Fig. 9a), understanding that the as grown samples always feature a much smaller conductivity if compared to those submitted to RTA. As a general rule, once again two competing phenomena must be taken into account to describe the system: 1) conductivity increases from VZO-2% to VZO-3% 2) crystallinity also influences conductivity, such that the samples submitted to RTA always behave better than the pristine ones. Results are summarized in the phase space plot of Fig. 9b, where *d*_*33*_ is plotted against the ratio between V^3+^ to V^5+^ V 2p_3/2_ peak after XPS (chemical properties) and the crystallinity represented by the (002) peak height after XRD (structural properties), both data taken from Table 2. As a general rule, we put in evidence that the lower the ratio and the higher the crystallinity, the higher the engineering performance of piezoelectricity. One may envisage that our results are close to the theoretical maximum performance output that can be extrapolated for the VZO, where the ratio V^3+^/V^5+^ is 1: *d*_*33,max*_ = 110 pm·V^−1^.

**Figure 10:**
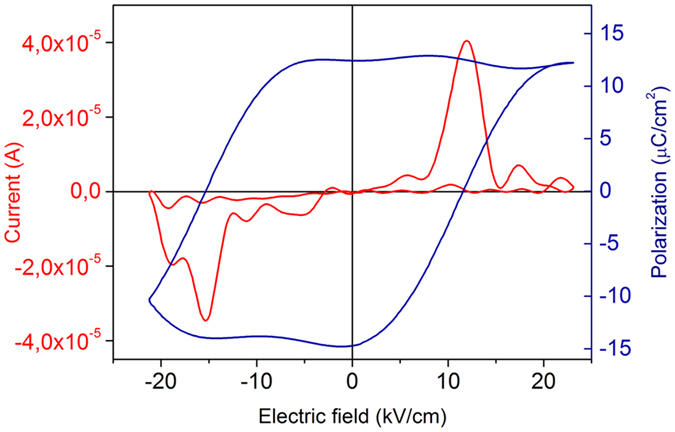
*I-V* and *P-E* hysteresis curve for sample VZO-2% after 5 min RTA.

The promising piezoelectric behavior observed for the sample VZO-2% after annealing for 5 min is also highlighted by the corresponding *I-V* characteristic and *P-E* curve shown in Fig. 10. The presence of switch current peaks, together with a closed-loop butterfly polarization curve, witnesses the presence of ferroelectric domains and their switching under the application of a sufficiently high electric field. To the best of our knowledge, this is the first evidence of a ferroelectric switching behavior in both the current and polarization curves at the same time, ever reported in the literature for V-doped ZnO. Some evidences from the literature, regarding the presence of a closed-loop polarization curve for V-doped ZnO nanostructures, have been already reported in the case of VZO nanorods grown using the hydrothermal approach^38^, where a remnant electrical polarization *P*_*r*_ of 4.83 μC·cm^−2^ was obtained. In the current work we succeeded in obtaining a higher *P*_*r*_ of 12.5 μC·cm^−2^, that supports the further investigation of VZO thin films as promising lead-free piezoelectric materials, which could be successfully integrated in energy harvesting systems and for a new generation of MEMS/NEMS devices. Additional efforts are still required in the optimization of VZO thin films so that *d*_*33*_ values in the order of 150–200 pm·V^−1^ could be reached. Only in such situation VZO films might become potentially comparable and competitive against sputtered PZT thin films.

The observed improvement of the piezoelectric response of V-doped ZnO thin films can be ascribed to different effects. The first one is the substitution of Zn^2+^ ions within the wurtzite structure by V^3+^ and V^5+^ ions, both having a higher positive charge than Zn^2+^. This results in V^3+^ –O and V^5+^ –O chemical bonds with a stronger polarity than Zn^2+^ –O ones, that can be easily aligned according to the direction of the applied electric field, hence inducing the piezoelectric mechanical strain. The corresponding stronger polarity induces a switchable spontaneous polarization. The second effect is due to the ease of rotation of V–O chemical bonds due to the substitution of Zn^2+^ ions by smaller V^5+^ ones. On the contrary, V^3+^ ions have a bigger ionic radius and do not further facilitate the bond rotation. Finally, all the annealed samples show a general improvement of the crystal structure. This results into a strong orientation along the *c*-axis direction that further promotes the piezoelectric response of the materials and increases their electrical conductivity.

A similar behavior has been already observed for other doping elements, such as Fe^28^. In that case, the effect of Fe^2+^ and Fe^3+^ ions on the piezoelectric properties was investigated. Only Fe^3+^ species, having a smaller size (0.64 Å) and a higher positive charge than Zn^2+^ resulted in effectively improving the piezoresponse of ZnO. On the contrary, bigger Fe^2+^ ions (0.76 Å), having the same positive charge as Zn^2+^, lowered the piezoelectric behavior of ZnO thin films, due to the difficult rotation of non-collinear Fe^2+^ –O chemical bonds induced by the larger ionic radius of Fe^2+^.

It is worth noting that, despite still showing a predominant presence of V^3+^ species even after annealing, some samples featured encouraging piezoelectricity as well. Therefore, the presence of V^5+^ species should be strongly promoted since resulting in better piezo/ferroelectric phenomena. However, it is found that also V^3+^ ions do not suppress piezoelectricity at all, but on the contrary generally improve the piezoelectric response with respect to the pristine ZnO case.

## Methods

### Deposition of Vanadium doped ZnO thin films

VZO thin films incorporating different amounts of V (2%, 2.5%, 3%) were prepared by radio-frequency (RF) magnetron sputtering (KS-300 Confocal Dual machine, Kenosistec) starting from a circular ZnO:V target (7.5 cm in diameter) prepared by mixing ZnO and NH_4_VO powders. After annealing at 500 °C for 1 h in air to decompose NH_4_VO_3_, powders were ball-milled and finally sintered by Spark Plasma Sintering method in vacuum, at 700 °C for 5 min. Suitable vacuum conditions with a base pressure of 1.7 × 10^−5^ Pa were obtained with a rotary and a turbo molecular pump. A RF signal at a working frequency of 13.56 MHz was employed to light the plasma. Each deposition process was carried out with a target-to-substrate distance of about 8 cm, at room temperature, with a RF power density of 1.76 W∙cm^−2^, and a fixed pressure of 1 Pa. In order to tune the final amount of V incorporated in the deposited thin films, VZO samples were prepared under different oxygen partial pressure conditions, ranging from 0% up to 10%^56^,^57^. In the following, each sample name will be labeled according to the corresponding V content. To prevent any incorporation of contaminants in the deposited film, the target was cleaned with a 15 min sputtering process in a pure Ar atmosphere before starting the depositions.

**Figure 11:**
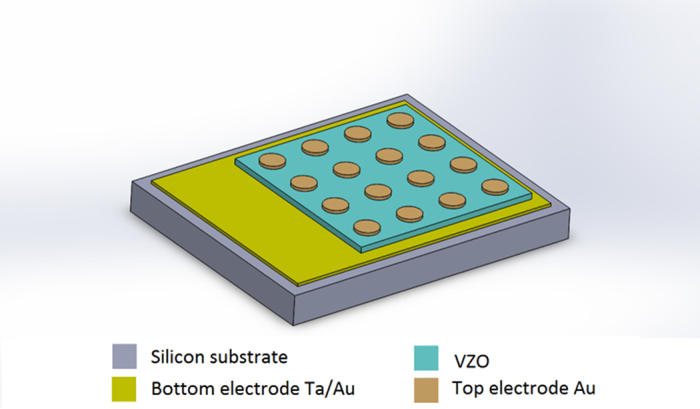
Scheme of the VZO-based devices deposited on Si/Ta/Au in the CPE configuration.

Si wafers and Si/Ta(10 nm)/Au(100 nm) were used as substrates. After each deposition, on some samples a Rapid Thermal Annealing (RTA) process was carried out, using a Solaris100 rapid thermal annealer from Surface Science Integration. The samples were heated at 600 °C (heating ramp rate 10 °C/min) for different times (1 min and 5 min) under continuous pure O_2_ flow (2 Standard Liters per Minute, SLM). For the evaluation of the piezoelectric properties, VZO thin films were coupled to a pair of metal electrodes, by adopting a cross-point electrode (CPE) structure. For this purpose, 200 nm-thick circular Au top electrodes were sputtered on the surface of the prepared VZO samples through a shadow mask with 2 mm diameter circular openings. A sketch of the final devices in the CPE configuration is shown in Fig. 11.

### Materials characterization

The average thickness of VZO thin films was investigated by Field Emission Scanning Electron Microscopy (FESEM), using a Zeiss Supra 40 microscope. X-ray diffraction (XRD) measurements were performed by a Panalytical X’Pert Pro Diffractometer in the Bragg-Brentano configuration, equipped with a Cu Kα radiation as X-ray source (λ = 1.540 59 Å). X-ray photoelectron spectroscopy (XPS) was carried out by using a PHI 5000 VersaProbe (Physical Electronics) system. The X-ray source was a monochromatic Al Kα radiation. Sputter cleaning has been performed using the Ar^+^ source with a 2 kV ions accelerating voltage (10 μA ion current) and 1 min sputtering time. The piezoelectric properties of VZO thin films were studied using an aixDBLI Double Beam Laser Interferometer system, from aixACCT Systems. A large signal excitation voltage was applied on the sample at room temperature, and the mechanical displacement induced on the piezoelectric thin film acquired by the optical components of the interferometer system located in a vibration damped chamber. The average piezoelectric coefficient *d*_*33*_ for each sample is estimated according to the law of converse piezoelectric effect^28^,^35^. Electrical characterization was performed using a Keithley 2635 A and a standard two point micro-contact setup, at room temperature in air.

## Additional Information

**How to cite this article:** Laurenti, M. *et al*. Lead-free piezoelectrics: V^3+^ to V^5+^ ion conversion promoting the performances of V-doped Zinc Oxide. *Sci. Rep.* **7**, 41957; doi: 10.1038/srep41957 (2017).

**Publisher's note:** Springer Nature remains neutral with regard to jurisdictional claims in published maps and institutional affiliations.

## Supplementary Material

Supplementary Information

## Figures and Tables

**Table 1:** 2θ position for (002) XRD peaks detected from the as-grown VZO thin films.

Sample name	(002) position	Zn at.%	O at.%	V at.%	HR V 2p_3/2_ area, %	
					V_2_O_3_	V_2_O_5_
VZO-2%	34.01°	51.2	46.8	2.0	71.3	28.7
VZO-2.5%	34.03°	51.0	46.5	2.5	86.5	13.5
VZO-3%	34.08°	50.8	46.2	3.0	71.9	28.1

Quantitative compositional XPS analysis and peak area under HR V 2p_3/2_ contribution for the different as-grown VZO thin films.

**Table 2:** Chemophysical properties of the prepared samples.

Sample name	HR V2p_3/2_ area, %			(002) peak height [A.U.]	*d*_*33*_ [pm·V^−1^]	Resistivity [Ω·cm]
	V_2_O_3_	V_2_O_5_	Ratio			
VZO-2%	71.3	28.7	2.5	0.27	23	269.0 ± 0.5
VZO-2% 1 min	71.8	28.2	2.5	0.60	65	26.25 ± 0.05
VZO-2% 5 min	65.3	34.7	1.9	1.00	85	0.903 ± 0.002
VZO-2.5%	86.5	13.5	6.4	0.20	9	(3.14 ± 0.03) × 10^7^
VZO-2.5% 1 min	80.7	19.3	4.2	0.55	10	1.096 ± 0.003
VZO-2.5% 5 min	77.9	22.1	3.5	0.55	51	13.32 ± 0.05
VZO-3%	71.9	28.1	2.6	0.08	6	(3.11 ± 0.02) × 10^7^
VZO-3% 1 min	83.3	16.7	5.0	0.12	25	0.295 ± 0.001
VZO-3% 5 min	76.7	23.3	3.3	0.19	44	4.160 ± 0.003

Peak area under HR V 2p_3/2_ contribution, (002) XRD spectra peak height, *d*_*33*_ and resistivity.
