# Supplementary material for: Lead-free piezoelectrics: V3+ to V5+ ion conversion promoting the performances of V-doped Zinc Oxide
Source: Sci Rep. 2017 Feb 6;7:41957. doi: 10.1038/srep41957 (PMC5292744; doi:10.1038/srep41957)
Supplement: Supplementary Information [file srep41957-s1.pdf]

Lead-free piezoelectrics: V^{3+} to V^{5+} ion conversion promoting the performances of V-doped Zinc Oxide

Marco Laurenti[1,2], Micaela Castellino[1], Denis Perrone[1], Abil Asvarov[1,3], Giancarlo Canavese[2], and Alessandro Chiolerio[1,]*

[1]Center for Sustainable Feautures, Istituto Italiano di Tecnologia, C.so Trento 21, 10129 Turin, Italy

[2]Department of Applied Science and Technology, Politecnico di Torino, C.so Duca degli Abruzzi 24, 10129 Turin, Italy

[3]Institute of Physics, Dagestan Scientific Center, Russian Academy of Sciences, Yaragskogo Str. 94, 367003 Makhackhala, Russia

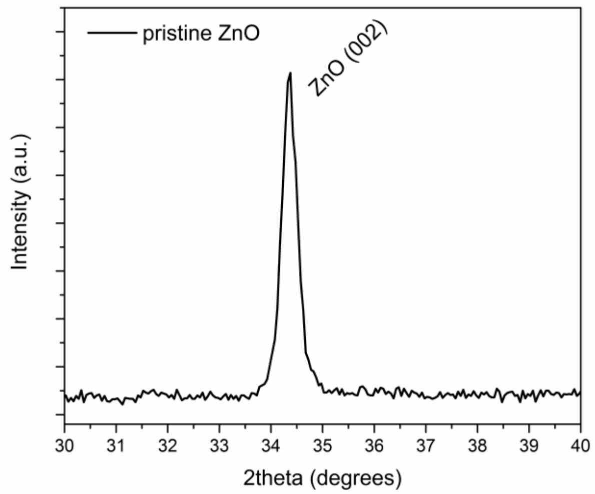

Figure S1. XRD pattern of pristine ZnO thin film.

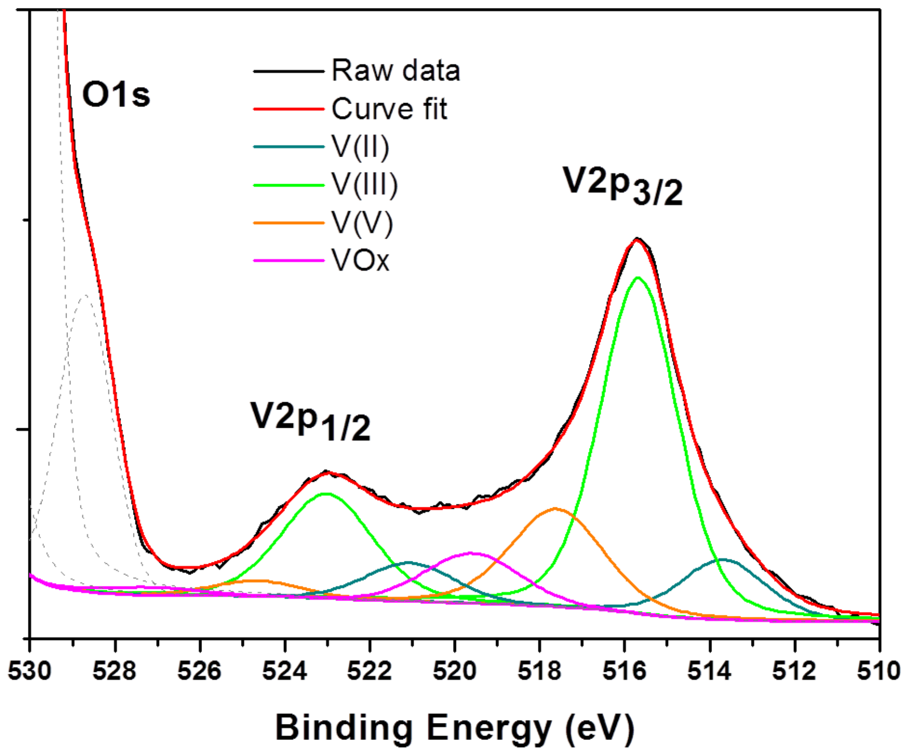

Figure S2. HR XPS spectrum related to O1s-V2p contribution, acquired from ZnO:V ceramic target.
